# CSF Neurofilament Light Chain Levels in Primary Progressive MS: Signs of Axonal Neurodegeneration

**DOI:** 10.3389/fneur.2018.01037

**Published:** 2018-12-14

**Authors:** Marc Pawlitzki, Stefanie Schreiber, Daniel Bittner, Julia Kreipe, Frank Leypoldt, Klemens Rupprecht, Roxana O. Carare, Sven G. Meuth, Stefan Vielhaber, Peter Körtvélyessy

**Affiliations:** ^1^Department of Neurology, Otto-von-Guericke University, Magdeburg, Germany; ^2^Department of Neurology with Institute of Translational Neurology, University Hospital of Muenster, Münster, Germany; ^3^German Center for Neurodegenerative Diseases, (DZNE), Magdeburg, Germany; ^4^Neuroimmunology, Institute of Clinical Chemistry, University Hospital Schleswig-Holstein, Kiel, Germany; ^5^Department of Neurology, Charité-Universitätsmedizin Berlin, Berlin, Germany; ^6^Faculty of Medicine, University of Southampton, Southampton, United Kingdom; ^7^German Center for Neurodegenerative Diseases (DZNE), Berlin, Germany

**Keywords:** multiple sclerois and neuroimmunology, neurofilament light chain (NFL), amyotrofic lateral sclerosis, primary progressive multiple sclerosis, cerebrospical fluid (CSF)

## Abstract

**Objectives:** Elevated neurofilament light chain (NFL) levels within the cerebrospinal fluid (CSF) are a biomarker representing axonal neurodegeneration in rapid progressive neurodegenerative diseases such as amyotrophic lateral sclerosis (ALS). It is unclear to what extent the levels of NFL increase in the CSF (CSF-NFL) in a chronic neuroinflammatory process with axonal neurodegeneration, as found in primary progressive multiple sclerosis (PPMS).

**Methods:** We used a multicenter approach to statistically compare CSF-NFL levels between PPMS patients (*n* = 50), ALS patients (*n* = 50), and healthy controls (*n* = 50). Clinical findings, including disease duration, expanded disability status scale (EDSS), electrophysiological recordings such as visual evoked potentials or spinal and cerebral MRI, and previously administered treatment were selected as experimental parameters retrospectively.

**Results:** Median [range] CSF-NFL concentrations in PPMS patients were significantly higher than in the controls [1724 (799–4275) pg/ml vs. 1202 (612–2934) pg/ml, *p* = 0.015], and significantly lower compared to ALS patients [1724 (799–4275) pg/ml vs. 10238 (2610–35138) pg/ml, *p* < 0.001]. There was no correlation between CSF-NFL and disease duration (*p* = 0.5), EDSS (*p* = 0.2) or treatment (*p* = 0.3).

**Conclusion:** We conclude that CSF-NFL may mirror the proposed slow axonal degeneration in PPMS, but does not reflect the disease severity.

## Introduction

The pattern underlying the concentrations of neurofilament light chain (NFL) in the cerebrospinal fluid (CSF), referred to as CSF-NFL, in diseases with slow progressive axonal degeneration, including primary progressive multiple sclerosis (PPMS), are not clear. Aggressive axonal injury after acute inflammatory events in patients with multiple sclerosis (MS), and rapidly progressive neurodegenerative diseases with a predominant affection of the central motor system such as amyotrophic lateral sclerosis (ALS), result in a clear pathological increase of the CSF-NFL levels (1–3). In contrast, studies of PPMS display a wide spectrum of CSF-NFL concentrations, including cases with low (< 500 pg/ml) (4, 5) or not detectable CSF-NFL concentrations (6), and cases with high CSF-NFL levels (>10,000 pg/ml) (7) comparable with the documented ranges in relapsing remitting MS disease courses (7–9), painting a heterogeneous picture (10, 11). Reasons may be the small sample sizes selected for these PPMS studies (6, 8) or the inclusion of patients with partially acute inflammatory disease activity and only occasional motor impairment, thus not the typical long-standing chronic disease progression (12).

Here we conducted a cross-sectional multicenter study measuring the CSF levels of the stable protein NFL (1) in patients suffering from mainly long-standing PPMS without acute inflammatory disease activity, and compared them to the CSF levels in ALS patients and disease controls.

## Materials and Methods

### Patients, Controls, and Clinical Assessment

Our study was approved by the local ethics committees in Magdeburg (No. 07/17) and Kiel (D525/16) and a general commitment of the Charité Berlin Clinic for external analyzes of retrospective data in line with an external German ethics vote. We included CSF samples from (i) *n* = 50 patients with clinically definite PPMS according to the McDonald criteria 2010 (13) recruited at the Departments of Neurology at the Otto-von-Guericke University Magdeburg, the Charité Berlin and the University Hospital Schleswig-Holstein, Kiel, Germany; (ii) *n* = 50 patients suffering from probable and definite ALS according to the revised El Escorial criteria (14) recruited at the Department of Neurology in Magdeburg; and (iii) *n* = 50 healthy controls (HC) recruited in Magdeburg, comprising cases with non-specific complaints who underwent lumbar puncture (LP) during a routine diagnostic examination conducted to rule out any neurological condition. None of the controls suffered from a neurological disorder (neuroinflammatory or neuromuscular), in particular not from MS, peripheral polyneuropathies, muscle or motor neuron disease, nor did they display any specific abnormalities during the neurological examination (15, 16). In addition to the clinical classification, patients included in the control group also fulfilled the following laboratory criteria defining a non-inflammatory CSF: < 5 cells/μl CSF, < 2 mM lactate in the CSF, no disruption of the blood/CSF barrier (defined by the albumin CSF/serum quotient), no oligoclonal bands (OCBs) in the CSF, an no intrathecal immunoglobulin (Ig)G, IgA, or IgM synthesis (17). All patients were retrospectively recruited between 2012 and 2017.

Clinical scoring [Expanded disability status scale (EDSS)] (18) was performed in close timely proximity to the LP. Electrophysiological measurements [(visual evoked potentials (VEP)] and cerebral as well as spinal magnetic resonance images were available for the patients from points in time close, but not identical to the performance of LP.

EDSS, T2-weighted MRI conducted close to the LP, and CSF levels were selected as parameters to verify the PPMS diagnosis, which was based on the clinical progression over the course of 1 year. These parameters were observed together with the existence of cerebral or spinal cord T2-intense lesions or oligoclonal bands (OCB) (13), and resulted in the following overall constellation: either patients showed evidence for (a) a dissemination in space (DIS) in the brain due to the existence of at least one T2-intense lesion found in at least one cerebral area characteristic for MS [periventricular, juxtacortical, or infratentorial; in 50 (100%) of the patients], or (b) a DIS in the spinal cord due to the existence of at least two T2-intense lesions [34 (68%)], or (c) CSF OCB [42 (84%)]. Moreover, neither did any of the PPMS patients present an enhanced T2 lesion load compared to previous diagnostic MRI scans, nor could any Gadolinium-enhanced cerebral or spinal cord T1-weighted MRI lesions be detected. Thus, the absence of MRI progression might mirror a mostly non-inflammatory phenotype of PPMS patients in line with previous investigations (19, 20).

VEP evaluations were documented to describe axonal integrity loss in PPMS according to previously defined diagnostic criteria of PPMS (13). Latencies of the P100 exceeding 2.5 SDs from normative data were considered as abnormal VEPs. Twenty PPMS patients revealed abnormal VEPs in both eyes, 11 patients showed abnormal VEPs in one eye only, and 13 patients had normal VEPs. For the remaining 6 patients, no data were available.

Disease duration was defined as the time in months between symptom onset and the LP.

### CSF Measures

For all PPMS patients, the LP was performed at each respective University, while for all ALS subjects and the healthy controls, the LP was conducted in Magdeburg. At each center, CSF cells were counted immediately after the LP and total protein, albumin quotient (Q_alb_) and oligoclonal bands (for PPMS patients only) were measured. Every sample was stored at −80°C and shipped on dry ice for CSF-NFL measurement. CSF-NFL levels were determined in Magdeburg using commercially available ELISA kits (UmanDiagnostics NF-light®, Umeå, Sweden, catalog number 10-7001 CE). Intraassay coefficient of variance is 7.4% and interassay coefficient of variance is 6% (21). Every measurement is performed together with a blank and a commercial positive and negative control provided by the manufacturer. Samples were measured in serial procedures and not in batches.

### Statistical Analysis

Statistical analysis was conducted using SPSS 21 (IBM). Groups were compared with respect to categorical (using a χ2-test) and continuous variables (using a *t*-test or Mann-Whitney U-test or a Kruskal–Wallis one-way analysis of variance (ANOVA) applying pairwise Dunn-Bonferroni *post-hoc* testing). Spearman's rank correlations were performed between CSF-NFL and age, between CSF-NFL and further CSF measures (cell count, protein, Q_alb_), as well as between CSF-NFL and clinical scores (EDSS). *P*-values < 0.05 were deemed to be statistically significant.

## Results

### Cohorts

The demographics, clinical and CSF data of the cohorts are shown in Table 1. There were no age or sex (*p* = 0.2) related differences between PPMS, ALS and HC, whereas the values for the CSF cell count, protein and Q_alb_ varied between the diagnostic groups Table 1. Mean [SD] disease duration was significantly longer in PPMS [86 (952) months] compared to ALS [15 (17) months, *p* < 0.001]. For PPMS, disability severity was high (median EDSS 6) at the time of the LP. The PPMS patients recruited from the three different University centers did not differ with respect to age, sex, CSF protein, oligoclonal band positivity, median EDSS or disease duration. There were, however, inter-center differences regarding the prevalence of spinal cord lesions (*p* = 0.017), and the values for CSF cell count and Q_alb._ Several PPMS patients, *n* = 11 (28%), received immunomodulatory and immunosuppressive therapy (“treatment attempt”), comprising Rituximab (*n* = 2), and intravenous (*n* = 3) or intrathecal (*n* = 6) methylprednisolone. The Berlin cohort contained significantly more treated patients than the Magdeburg and Kiel cohorts (*p* = 0.001) Table 1.

**Table 1:** N, number of participants; unless otherwise reported mean [standard deviation] (range) is given.

	**PPMS (*N* = 50)**	**ALS (*N* = 50)**	**HC** **(*N* = 50)**	**PPMS study centers**			***P*****-values**					
				**MD** **(*N* = 25)**	**Berlin (*N* = 12)**	**Kiel (*N* = 13)**	**MD vs. Berlin**	**MD vs. Kiel**	**Berlin vs. Kiel**	**PPMS vs. HC**	**PPMS vs. ALS**	**ALS vs. HC**
Age at lumbar puncture (years)	53 [11] (25–77)	54 [10] (33–74)	53 [11] (34–76)	51 [11] (25–71)	57 [13] (35–77)	55 [10] (36–73)	0.3	0.3	0.3	0.8	0.8	0.8
Male sex, *N* (%)	29 (58)	33 (66)	24 (48)	17 (68)	7 (58)	5 (39)	–	–	–	–	–	–
Disease duration (months)	86 [92] (2–94)	–	–	86 [73] (6–255)	92 [90] (2–353)	82 [128] (10–494)	0.5	0.5	0.5	–	–	–
Median EDSS	6 (2–8)	–	–	5 (2–7.5)	6 (3–8)	4.5 (3–8)	0.3	0.3	0.3	–	–	–
≥1 Spinal cord lesions *N* (%)	34 (68)	–	–	17 (68)	5 (42)	12 (92)	–	–	–	–	–	–
≥1 cerebral T2 lesion *N* (%)	50 (100)	–	–	25 (100)	12 (100)	13 (100)	–	–	–	–	–	–
Pathological VEP, *N* (%)	31 (62)	–	–	18 (72)	6 (50)	7 (54)	–	–	–	–	–	–
Treatment, *N* (%)	11 (22)	–	–	1 (4)	10 (83)	0 (0)	–	–	–	–	–	–
Median CSF Cell count /μl	2.5 (0–131)	1 (0–6)	1 (0–4)	3 (0–14)	2 (0–29)	4 (1–131)	1.0	**0.02**	0.3	** < 0.001**	**0.001**	1.0
Median CSF protein (mg/dl)	441 (236–1047)	480 (187–1240)	351 (179–616)	407 (248–1047)	451 (236–767)	517 (312–875)	0.08	0.08	0.08	**0.001**	1.0	** < 0.001**
Positive OCB, *N* (%)	42 (84)	0 (0)	0 (0)	24 (96)	9 (75)	9 (69)	0.1	0.1	0.1	–	–	–
Median Q_alb_	6.0 (2.0–18.8)	6.8 (2.4–16.6)	4.8 (1.5–10.5)	5.0 (2.0–18.8)	6.8 (4.2–12.6)	8.2 (3.2–15.2)	0.2	**0.030**	1.0	**0.016**	1.0	**0.003**
Median CSF-NFL (pg/ml)	1724 (799–4275)	10238 (2610–35138)	1202 (612–2934)	1668 (990–4275)	1591 (877–2557)	2016 (799–4158)	0.5	0.5	0.5	**0.015**	** < 0.001**	** < 0.001**

*ALS, amyotrophic lateral sclerosis; CSF, cerebrospinal fluid; EDSS, Expanded Disability Status Scale; HC, healthy controls; MD, Magdeburg; NFL, neurofilament light chain; OCB, oligoclonal bands; PPMS, primary progressive multiple sclerosis; Q_alb_, albumin quotient; VEP, visual evoked potential. Disease duration was defined as the timespan between symptom onset and the date of lumbar puncture. For group comparisons a χ2-test or a Kruskal-Wallis one-way analysis of variance with post-hoc Dunn-Bonferroni-testing were conducted. P-values < 0.05 were deemed to be statistically significant (Bold)*.

## CSF-NFL

When considering the whole sample, we found small- to medium-effect size correlations between CSF-NFL and age (rho = 0.2, *p* = 0.03), Q_alb_ (rho = 0.3, *p* < 0.001), and CSF protein (rho = 0.4, *p* < 0.001), while there was no correlation with sex (Z = −0.4, *p* = 0.7) or CSF cell count (rho = 0.01, *p* = 1.0).

**Figure 1:**
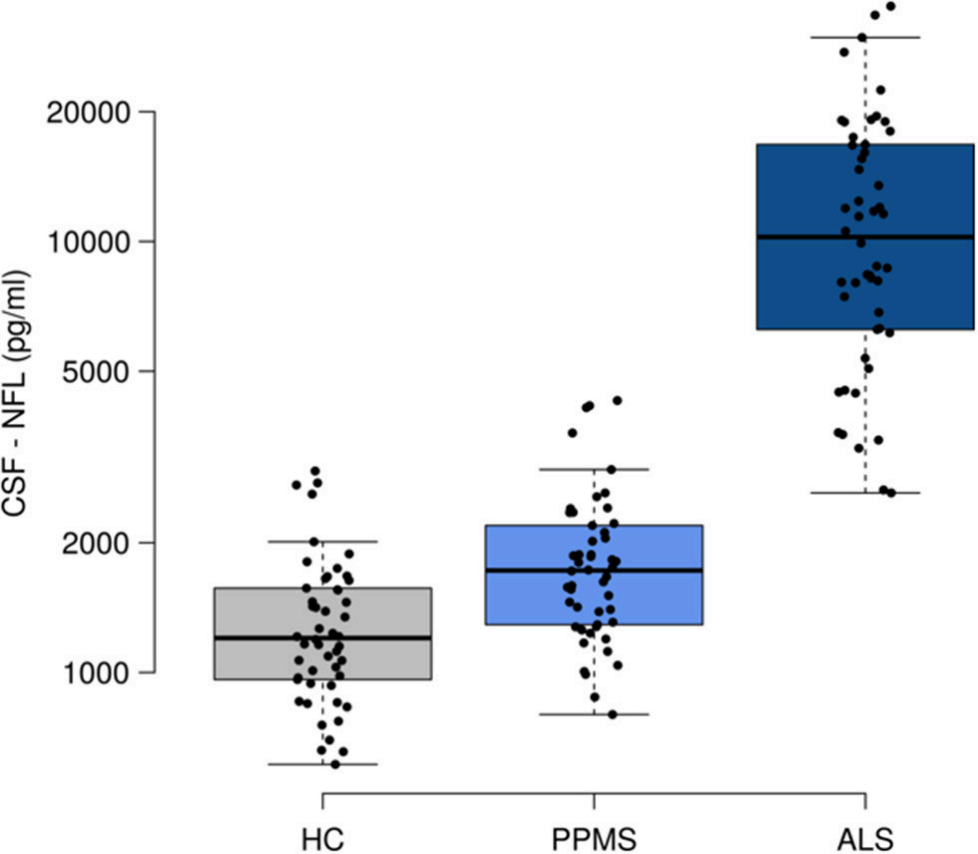
Neurofilament light (NFL) in cerebrospinal fluid (CSF). ALS, amyotrophic lateral sclerosis; HC, healthy controls; PPMS, primary progressive multiple sclerosis. Boxes indicates the interquartile range, bars indicates median CSF-NFL values, and whiskers present the 95% Cl. The dots present the individual values. Group comparisons were conducted using a Kruskai-Wallis one-way analysis of variance with *post-hoc* Dunn-Bonferroni-testing. *P*-values < 0.05 were deemed to be statistically significant. PPMS and ALS patients showed higher CSF-NFL levels than HC, while ALS had higher NFL values than PPMS.

**Figure 2:**
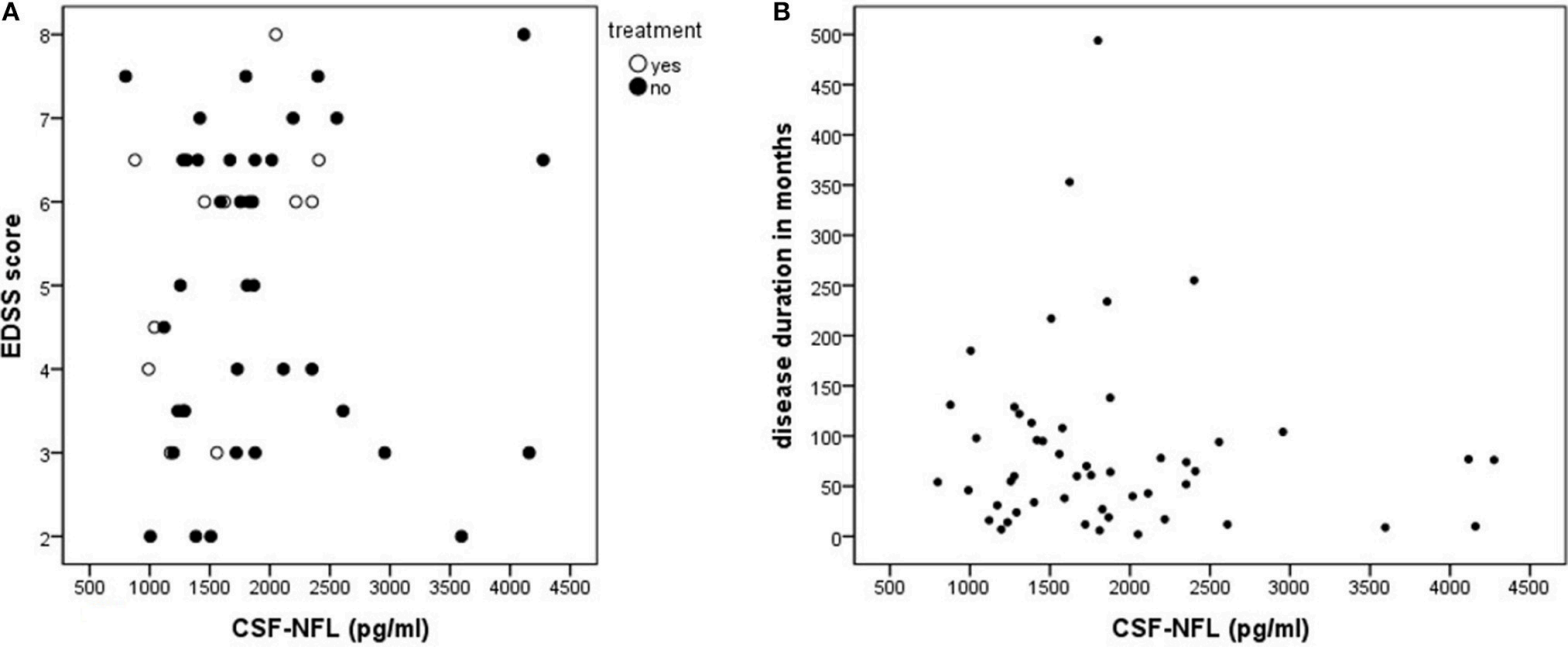
**(A,B)** The distribution of neurofilament light (NFL) levels in cerebrospinal fluid (CSF) of primary progressive multiple sclerosis patients. EDSS, Expanded disability status scale; HC, healthy controls; PPMS, primary progressive multiple sclerosis. Figure 2A shows the missing relationship between the CSF-NFL concentration and clinical state (EDSS) and current treatment. The CSF-NFL level. Figure 2B illustrates the missing correlation between CSF-NFL and disease duration.

There was a significant group effect of CSF-NFL levels when comparing PPMS to ALS (*p* < 0.001), and *post-hoc* analysis revealed lower median [range] levels for PPMS compared to ALS [1724 (799–4275) pg/ml vs. 10238 (2610–35138) pg/ml, *p* < 0.001], ALS compared to HC [1202 (612–2934) pg/ml, *p* < 0.001] and PPMS compared to HC (*p* = 0.015) Figure 1. In PPMS, CSF-NFL concentrations did not differ between treated or untreated patients (z = −1.0, *p* = 0.3) Figure 2, between patients with completely normal VEPs or only one eye with pathological VEPs (rho = −1.9, *p* = 0.05), and also not between study centers (*p* = 0.5); CSF-NFL concentrations were not related to disease duration (rho = −0.9, *p* = 0.5) or EDSS (rho = 0.17, *p* = 0.2) Figure 2.

## Discussion

We investigated CSF-NFL levels in clinically definite PPMS patients suffering from a mainly long-standing disease without inflammatory disease activity. To our knowledge this is the largest cohort of patients investigated to date addressing the question whether CSF-NFL levels can mirror disease associated axonal neurodegeneration and its value as a clinical biomarker in PPMS patients. Our analysis reveals that PPMS diagnosis relates to significantly different levels of CSF-NFL than the levels found for the other two groups, and affirms a moderate PPMS CSF-NFL level situated in-between values for HC and ALS patients.

Since several studies have shown contradicting results of lower (1) or higher CSF-NFL levels (22, 23) in PPMS compared to relapsing remitting MS, the degree of chronic axonal loss in the absence of acute inflammatory activity remains unclear. In line with previous results, we found significantly elevated CSF-NFL values in PPMS compared to HC (1, 6, 22, 24). The previously published studies comprised smaller PPMS samples, or included patients displaying inflammatory disease activity and short-lasting disease symptoms leading us to question whether a common PPMS disease course was observed. We addressed the shortcomings of former studies by taking a considerably large PPMS cohort into account, characterized by a primary chronic progressive disease course mainly with long-lasting symptoms.

The subjects included in our PPMS cohort are likely to suffer from corticospinal tract (CST) demyelination, typically found in long-standing PPMS (12). This is supported by the high median EDSS, reflecting manifest motor impairment, and the considerably great prevalence of spinal cord lesions in our PPMS cohort Table 1 NFL is a cytoskeleton protein, that is highly abundant in large-caliber myelinated axons found abundantly in the CST (25–27). A former study using diffusion tensor imaging (DTI) has shown that an increase in CSF-NFL reflects CST degeneration in ALS (28). We thus assume that CSF-NFL elevation in PPMS particularly mirrors chronic CST degeneration, in addition to cortical thinning (29).

We conducted a direct CSF-NFL comparison between PPMS and ALS, with both diseases sharing clinical signs resulting from the degeneration of the long cerebral and spinal cord tracts in the central motor system (10). These commonalities could be reflected by significantly elevated CSF-NFL concentrations for both PPMS and ALS patients compared to healthy controls. However, ALS patients have a shorter disease duration than PPMS patients, with ALS being characterized by a more rapid and aggressive neurodegenerative disease course relating to a median survival time of 36 months (30), compared to 14–33 years in PPMS (31). The corresponding neuroaxonal damage and depletion in ALS is presumed to take place in a more accelerated manner, potentially explaining the CSF-NFL group differences between PPMS and ALS (10). The predominant demyelination of axons with still partially intact cytoskeleton and neurons in PPMS could also explain the CSF-NFL group differences between PPMS and ALS (10). Moreover, in PPMS, a stronger CSF-NFL level increase was documented at the disease onset, suggesting a greater progression of neuroaxonal degeneration in early and inflammatory active disease stages compared to the long-standing disease pattern (22, 32).

Moreover, when considering our previous work on CSF-NFL, PPMS patients presented significantly lower levels not only compared to ALS patients but also compared to patients with a slow neurodegenerative disease like frontotemporal dementia (FTD) (21), emphasizing the differences between primary neurodegeneration (slow: FTD, rapid: ALS) and inflammatory driven neuro-axonal injury (PPMS).

In line with recent PPMS studies we did not find any relationship between the clinical impact, as measured using the EDSS, and CSF-NFL (1, 7, 8). The concentrations of CSF-NFL are considered to reflect more cumulative underlying pathological processes that are not captured by disability scores at any given time, especially since slow progressive PPMS rarely presents a disease course with acute clinical worsening or dynamic changes in the EDSS score (33). Accordingly, those studies showing a—modest—correlation between increased CSF-NFL levels and clinical function mainly included patients at initial disease stages with acute clinical exacerbations (6, 24), or patients with relapsing remitting MS (24). The lack of relationship between NFL and VEP could be explained by the fact, that the abnormal VEP describes a localized, circumscribed axonal injury far away from lumbar CSF, which might not trigger the release of sufficient amounts of NFL in contrast to spinal injury (27, 34).

The PPMS patients recruited at the three different Universities differed with respect to (i) CSF cell count and album quotient, which were greater in the Kiel cohort, and (ii) treatment frequency, which was highest in the Berlin cohort. These findings lead us to assume that the Kiel patients display more (clinically silent) inflammatory activity (without corresponding relapses and MRI contrast-enhancing lesions), and we could also deduct that the Berlin University regularly applies off-label drugs in accordance with positive case series reported by the Berlin group (19, 35). However, the above parameters did not affect the PPMS patients' CSF-NFL levels, which did not differ between (i) the centers or (ii) treated and untreated cases.

Limitations of the present study include the absence of MRI measurements obtained exactly at the same point in time as the LP was performed. We therefore cannot completely exclude the presence of Gd-enhancing lesions. Moreover, longitudinal studies are needed to evaluate if NFL is a suitable biomarker to predict and adjudicate disability progression in PPMS. In addition, future studies should address the questions (i) if CSF-NFL could also aid to differentiate potential PPMS disease mimics, e.g., hereditary spastic paraplegia, and (ii) if composite measurement of DTI CST involvement, viewed together with CSF-NFL levels, may provide a better correlate with the overall functional state of PPMS patients. A further point of interest would be to measure the contribution of the inflammatory vs. neurodegenerative components influencing the CSF-NFL levels, through contrasting PPMS patients with acute inflammatory (MRI-) disease activity with those patients in progressive disease stages and the possible relations to therapeutic approaches.

In our current study we were able to demonstrate that CSF-NFL levels can clearly discriminate between slowly progressive neuroinflammatory (PPMS) and more rapid neurodegenerative (ALS) processes, but do not correlate with assessments of PPMS severity on a clinical disease scale. We conclude, that CSF-NFL mirrors the inflammation driven neurodegenerative aspect in MS and thus, CSF-NFL might not be an ideal biomarker in PPMS.

## Ethics Statement

We confirm that we have read the Journal's position on issues involved in ethical Publication and affirm that this report is consistent with those guidelines.

## Author Contributions

MP has access to all the data and takes responsibility for the data, accuracy of the data analysis, and interpretation of the data and drafting the manuscript for intellectual content. SS, DB, JK, FL, KR, SM, SV Design and conceptualization of the study; revising the manuscript for intellectual content; RC Design and conceptualization of the study; revising the manuscript for intellectual content and language improvement. PK Design and conceptualization of the study; revising the manuscript for intellectual content, Study supervision.

### Conflict of Interest Statement

MP received speaker honoraria from Roche, Genzyme and Novartis and travel/accommodation/meeting expenses from Novartis, Biogen Idec, Genzyme and MERCK Serono. KR has received research support from the German Ministry of Education and Research (BMBF/KKNMS, Competence Network Multiple Sclerosis) Merck Serono and Novartis as well as speaking fees and travel grants from Guthy Jackson Charitable Foundation, Bayer Healthcare, Biogen Idec, Merck Serono, Sanofi-aventis/Genzyme, Teva Pharmaceuticals, Roche and Novartis. SM receives honoraria for lecturing, and travel expenses for attending meetings from Almirall, Amicus Therapeutics Germany, Bayer Health Care, Biogen, Celgene, Diamed, Genzyme, MedDay Pharmaceuticals, Merck Serono, Novartis, Novo Nordisk, ONO Pharma, Roche, Sanofi-Aventis, Chugai Pharma, QuintilesIMS and Teva. His research is funded by the German Ministry for Education and Research (BMBF), Deutsche Forschungsgesellschaft (DFG), Else Kröner Fresenius Foundation, German Academic Exchange Service, Hertie Foundation, Interdisciplinary Center for Clinical Studies (IZKF) Muenster, German Foundation Neurology and Almirall, Amicus Therapeutics Germany, Biogen, Diamed, Fresenius Medical Care, Genzyme, Merck Serono, Novartis, ONO Pharma, Roche, and Teva. The remaining authors declare that the research was conducted in the absence of any commercial or financial relationships that could be construed as a potential conflict of interest.

## References

[1] Kuhle J Plattner K Bestwick JP Lindberg RL Ramagopalan SV Norgren N. A comparative study of CSF neurofilament light and heavy chain protein in MS. Mult Scler. (2013) 19:1597–603. 10.1177/1352458513482374 23529999

[2] Steinacker P Feneberg E Weishaupt J Brettschneider J Tumani H Andersen PM. Neurofilaments in the diagnosis of motoneuron diseases: a prospective study on 455 patients. J Neurol Neurosurg Psychiatr. (2016) 87:12–20. 10.1136/jnnp-2015-311387 26296871

[3] Gaiani A Martinelli I Bello L Querin G Puthenparampil M Ruggero S. Diagnostic and prognostic biomarkers in amyotrophic lateral sclerosis: neurofilament light chain levels in definite subtypes of disease. JAMA Neurol. (2017). 74:525–32. 10.1001/jamaneurol.2016.5398 28264096 PMC5822207

[4] Norgren N Sundström P Svenningsson A Rosengren L Stigbrand T Gunnarsson M. Neurofilament and glial fibrillary acidic protein in multiple sclerosis. Neurology (2004) 63:1586–90. 10.1212/01.WNL.0000142988.49341.D1 15534240

[5] Trentini A Comabella M Tintoré M Koel-Simmelink Marleen JA Killestein J Roos B. N-acetylaspartate and neurofilaments as biomarkers of axonal damage in patients with progressive forms of multiple sclerosis. J Neurol. (2014) 261:2338–43. 10.1007/s00415-014-7507-4 25228004

[6] Salzer J Svenningsson A Sundström P. Neurofilament light as a prognostic marker in multiple sclerosis. Mult Scler. (2010) 16:287–92. 10.1177/1352458509359725 20086018

[7] Teunissen CE Iacobaeus E Khademi M Brundin L Norgren N Koel-Simmelink MJA. Combination of CSF N-acetylaspartate and neurofilaments in multiple sclerosis. Neurology (2009) 72:1322–9. 10.1212/WNL.0b013e3181a0fe3f 19365053

[8] Malmeström C Haghighi S Rosengren L Andersen O Lycke J. Neurofilament light protein and glial fibrillary acidic protein as biological markers in MS. Neurology (2003) 61:1720–5. 10.1212/01.WNL.0000098880.19793.B6 14694036

[9] Modvig S Degn M Sander B Horwitz H Wanscher B Sellebjerg F. Cerebrospinal fluid neurofilament light chain levels predict visual outcome after optic neuritis. Mult Scler. (2016) 22:590–8. 10.1177/1352458515599074 26283696

[10] Abdelhak A Junker A Brettschneider J Kassubek J Ludolph AC Otto M. Brain-specific cytoskeletal damage markers in cerebrospinal fluid: is there a common pattern between amyotrophic lateral sclerosis and primary progressive multiple sclerosis? Int J Mol Sci. (2015) 16:17565–88. 10.3390/ijms160817565 26263977 PMC4581209

[11] Barro C Leocani L Leppert D Comi G Kappos L Kuhle J. Fluid biomarker and electrophysiological outcome measures for progressive MS trials. Mult Scler. (2017) 23:1600–13. 10.1177/1352458517732844 29041870

[12] Koch MW Greenfield J Javizian O Deighton S Wall W Metz LM. The natural history of early versus late disability accumulation in primary progressive MS. J Neurol Neurosurg Psychiatr. (2015) 86:615–21. 10.1136/jnnp-2014-307948 25091366

[13] Polman CH Reingold SC Banwell B Clanet M Cohen JA Filippi M. Diagnostic criteria for multiple sclerosis: 2010 revisions to the McDonald criteria. Ann Neurol. (2011) 69:292–302. 10.1002/ana.22366 21387374 PMC3084507

[14] Brooks BR Miller RG Swash M Munsat TL. El Escorial revisited: revised criteria for the diagnosis of amyotrophic lateral sclerosis. Amyotroph Lateral Scler Other Motor Neuron Disord. (2000) 1:293–9. 10.1080/146608200300079536 11464847

[15] Huchtemann T Körtvélyessy P Feistner H Heinze HJ Bittner D. Progranulin levels in status epilepticus as a marker of neuronal recovery and neuroprotection. Epilepsy Behav. (2015) 49:170–2. 10.1016/j.yebeh.2015.06.022 26211941

[16] Schreiber S Debska-Vielhaber G Abdulla S Machts J Schreiber F Kropf S Peripheral nerve atrophy together with higher cerebrospinal fluid progranulin indicate axonal damage in amyotrophic lateral sclerosis. Muscle Nerve (2017) 52:273–278. 10.1002/mus.25682 28472860

[17] Reiber H Lange P. Quantification of virus-specific antibodies in cerebrospinal fluid and serum: sensitive and specific detection of antibody synthesis in brain. Clin Chem. (1991) 37:1153–60. 1855284

[18] Kurtzke JF. Rating neurologic impairment in multiple sclerosis: an expanded disability status scale (EDSS). Neurology (1983) 33:1444–52. 10.1212/WNL.33.11.1444 6685237

[19] Hawker K O'Connor P Freedman MS Calabresi PA Antel J Simon J. Rituximab in patients with primary progressive multiple sclerosis: results of a randomized double-blind placebo-controlled multicenter trial. Ann Neurol. (2009) 66:460–71. 10.1002/ana.21867 19847908

[20] Montalban X Hauser SL Kappos L Arnold DL Bar-Or A Comi G. Ocrelizumab versus placebo in primary progressive multiple sclerosis. N Engl J Med. (2017) 376:209–20. 10.1056/NEJMoa1606468 28002688

[21] Körtvelyessy P Heinze HJ Prudlo J Bittner D. CSF biomarkers of neurodegeneration in progressive non-fluent aphasia and other forms of frontotemporal dementia: clues for pathomechanisms? Front Neurol. (2018) 9:504. 10.3389/fneur.2018.00504 30013506 PMC6036143

[22] Semra YK Seidi OA Sharief MK. Heightened intrathecal release of axonal cytoskeletal proteins in multiple sclerosis is associated with progressive disease and clinical disability. J Neuroimmunol. (2002) 122:132–9. 10.1016/S0165-5728(01)00455-6 11777552

[23] Madeddu R Farace C Tolu P Solinas G Asara Y Sotgiu MA. Cytoskeletal proteins in the cerebrospinal fluid as biomarker of multiple sclerosis. Neurol Sci. (2013) 34:181–6. 10.1007/s10072-012-0974-4 22362332

[24] Lycke JN Karlsson JE Andersen O Rosengren LE. Neurofilament protein in cerebrospinal fluid: a potential marker of activity in multiple sclerosis. J Neurol Neurosurg Psychiatr. (1998) 64:402–4. 10.1136/jnnp.64.3.402 9527161 PMC2170011

[25] Petzold A. Neurofilament phosphoforms: surrogate markers for axonal injury, degeneration and loss. J Neurol Sci. (2005) 233:183–98. 10.1016/j.jns.2005.03.015 15896809

[26] Petzold A Gveric D Groves M Schmierer K Grant D Chapman M. Phosphorylation and compactness of neurofilaments in multiple sclerosis: indicators of axonal pathology. Exp Neurol. (2008) 213:326–35. 10.1016/j.expneurol.2008.06.008 18619438 PMC2583254

[27] Kuhle J Gaiottino J Leppert D Petzold A Bestwick JP Malaspina A. Serum neurofilament light chain is a biomarker of human spinal cord injury severity and outcome. J Neurol Neurosurg Psychiatr. (2015) 86:273–9. 10.1136/jnnp-2013-307454 24935984

[28] Menke RAL Gray E Lu C-H Kuhle J Talbot K Malaspina A. CSF neurofilament light chain reflects corticospinal tract degeneration in ALS. Ann Clin Transl Neurol. (2015) 2:748–55. 10.1002/acn3.212 26273687 PMC4531057

[29] Barro C Benkert P Disanto G Tsagkas C Amann M Naegelin Y Serum neurofilament as a predictor of disease worsening and brain and spinal cord atrophy in multiple sclerosis. Brain (2018) 141:2382–91. 10.1093/brain/awy154 29860296

[30] Knibb JA Keren N Kulka A Leigh PN Martin S Shaw CE. A clinical tool for predicting survival in ALS. J Neurol Neurosurg Psychiatr. (2016) 87:1361–7. 10.1136/jnnp-2015-312908 27378085 PMC5136716

[31] Cottrell DA Kremenchutzky M Rice GP Koopman WJ Hader W Baskerville J. The natural history of multiple sclerosis: a geographically based study. 5. The clinical features and natural history of primary progressive multiple sclerosis. Brain (1999) 122 (Pt 4):625–39. 10.1093/brain/122.4.625 10219776

[32] Axelsson M Malmeström C Gunnarsson M Zetterberg H Sundström P Lycke J. Immunosuppressive therapy reduces axonal damage in progressive multiple sclerosis. Mult Scler. (2014) 20:43–50. 10.1177/1352458513490544 23702432

[33] Mañé-Martínez MA Olsson B Bau L Matas E Cobo-Calvo Á Andreasson U. Glial and neuronal markers in cerebrospinal fluid in different types of multiple sclerosis. J Neuroimmunol. (2016) 299:112–7. 10.1016/j.jneuroim.2016.08.004 27725108

[34] Giovannoni G. Multiple sclerosis cerebrospinal fluid biomarkers. Dis Markers (2006) 22:187–96. 10.1155/2006/509476 17124340 PMC3851677

[35] Abu-Mugheisib M Benecke R Zettl UK. Repeated intrathecal triamcinolone acetonide administration in progressive multiple sclerosis: a review. Mult Scler Int. (2011) 2011:219049. 10.1155/2011/219049 22096630 PMC3196978

